# O066. Kynurenine pathway metabolites in cluster headache

**DOI:** 10.1186/1129-2377-16-S1-A87

**Published:** 2015-09-28

**Authors:** Martina Curto, Luana Lionetto, Matilde Capi, Andrea Negro, Lidia D'Alonzo, Ferdinando Nicoletti, Paolo Martelletti

**Affiliations:** 1 Department of Psychiatry, McLean Hospital, Harvard University, Boston, (MA) USA; 2 Advanced Molecular Diagnostics Unit, Sant'Andrea Hospital, Rome, Italy; 3 Department of Clinical and Molecular Medicine, Sapienza University of Rome, Rome, Italy; 4 I.R.C.C.S. Neuromed, Pozzilli, (IS) Italy

Cluster headache is a severe, disabling disorder with pain that ranks among the most severe known to humans. It is associated with accompanying autonomic symptoms ipsilateral to the pain and a sense of restlessness or agitation. Patients with cluster headaches have few therapeutic options, and a further 10-20% develop drug-resistant attacks. The often brief duration of cluster attacks makes abortive therapy a challenge, and preventive medications are almost always provided to patients.

Although NMDA-R activation by glutamate has been hypothesized to play a role in the pathophysiology of primary headache disorders, its role is still not fully understood. In fact, the trigeminovascular nociceptive transmission from primary afferents through the trigeminal nucleus caudalis and on to other parts of the CNS involves both the NMDA and non-NMDA glutamate receptors. The kynurenine pathway (KP), accounting for more than 90% of the tryptophan metabolism, generates neuroactive compounds that are able to interact with glutamate receptors both in the central and in the peripheral nervous systems. Among the KP metabolites, Kynurenic Acid (KYNA) and Quinolinic Acid (QUINA) have been shown to interact with ionotropic glutamate receptors. QUINA acts as an orthosteric agonist at the GluN2 subunits of NMDA receptors and it might regulate endogenous glutamate release and uptake inhibition and lipid peroxidation. In contrast, KYNA acts as a competitive antagonist at the glycine site on the GluN1 subunit of NMDA receptors, thereby inhibiting NMDA receptor function. KYNA also inhibits the kainate and the amino-3-hydroxy-5-methyl-4-isoxazole propionic acid (AMPA) glutamate receptors, and has a non-competitive inhibitory action on the a7-nicotinic acetylcholine receptors[1].

In this preliminary study we enrolled 14 cluster headache patients (CH, 13 males) and 15 age-matched healthy controls (HC, 14 males). We developed a HPLC tandem mass spectrometry method to assess the serum concentrations of KYNA, QUINA, Anthranilic acid and Kynurenine. Kynurenine serum levels resulted not significantly different between the groups (HC 0.33±0.1 and CH 0.35±0.14; Z= -0.53, p = 0.597), while both QUINA, KYNA and Anthranilic Acid were significantly reduced in cluster headache patients with respect to healthy controls (QUINA: HC 18.94±5.24 and CH 3.14±4.87; Z= -4.38, p < 0.001; KYNA: HC 3.53±1.33 and CH 2.53±1.20; Z= -12.45, p = 0.041; Anthranilic Acid: HC 1.28±1.14 and CH 0.17±0.06; Z= -4.46, p < 0.001). These results highlight that the endogenous regulation of the glutamatergic transmission in cluster headache might play an important role in its pathophysiology.

Written informed consent to publication was obtained from the patient(s).
